# Iatrogenic diaphragmatic hernia as a late complication of laparoscopic excisional biopsy of peritoneal nodules: A case report

**DOI:** 10.1016/j.ijscr.2020.01.044

**Published:** 2020-02-06

**Authors:** Kyoichi Hashimoto, Kazutaka Obama, Shigeru Tsunoda, Shigeo Hisamori, Tatsuto Nishigori, Masazumi Sakaguchi, Yoko Ueda, Nozomu Nakanishi, Yoshiharu Sakai

**Affiliations:** a Department of Surgery, Graduate School of Medicine, Kyoto University, Kyoto, 606-8507, Japan; b Department of Surgery, Uji-Tokushukai Medical Center, Uji, 611-0042, Japan

**Keywords:** Iatrogenic diaphragmatic hernia, Diagnostic laparoscopy, Soft coagulation

## Abstract

**Introduction:**

Iatrogenic diaphragmatic hernias have been reported as a rare complication of abdominal surgery, and only a few reports have suggested minimal intraoperative injury to the diaphragm as a cause.

**Presentation of case:**

We report a rare case of delayed diaphragmatic hernia caused by laparoscopic excisional biopsy of a peritoneal lesion on the diaphragmatic peritoneum. A 70-year-old woman with gastric gastrointestinal stromal tumor underwent laparoscopic partial gastrectomy and excisional biopsy for white nodules on the left diaphragmatic peritoneum using laparoscopic scissors. Slight bleeding that occurred after excisional biopsy was stopped using a soft-coagulation system. Six months after her first surgery, a left diaphragmatic defect and transverse colon migration into the thoracic cavity were detected on computed tomography. The patient subsequently underwent laparoscopic diaphragmatic hernia repair. The site at which the diaphragm defect occurred was consistent with the site of excisional biopsy. The defect was closed with interrupted suturing using non-absorbable threads, following which a composite mesh was applied above the suture site for reinforcement.

**Discussion:**

This is the first report of an iatrogenic diaphragmatic hernia after diagnostic laparoscopy for peritoneal excision biopsy of the diaphragmatic surface. In the present case, the coagulation procedure for hemostasis after peritoneal resection was considered to be the cause of the diaphragmatic hernia. The heat generated by soft coagulation might have unexpectedly reached and damaged the deep areas of the diaphragmatic muscle.

**Conclusion:**

Surgeons performing laparoscopic excisional biopsy of the diaphragmatic peritoneum should consider the potential risk for iatrogenic diaphragmatic hernias.

## Highlights

• We report a rare case of delayed diaphragmatic hernia caused by laparoscopic biopsy.
• Coagulation for hemostasis after biopsy could cause diaphragmatic hernia.
• The heat by soft coagulation might unexpectedly damage diaphragmatic muscle.

## 1 Introduction

Diagnostic laparoscopy has become a widespread modality for the diagnosis of gastrointestinal (GI) neoplasms, especially advanced gastric cancer. One of its major purposes has been the detection of small peritoneal disseminations of GI neoplasms, which are undetectable with routine imaging modalities [1,2]. Moreover, any observable peritoneal, intestinal, and mesenteric metastases are examined using diagnostic laparoscopy. For suspicious nodules, excisional biopsy is performed to detect peritoneal metastasis. Fewer complications have been reported using diagnostic laparoscopy than using other major surgeries [3].

Iatrogenic diaphragmatic hernias have been reported as a rare complication of abdominal surgery [[4], [5], [6], [7]], and a few reports have suggested minimal intraoperative injury to the diaphragm as a cause. Here we report a rare case of delayed iatrogenic diaphragmatic hernia due to possible diaphragmatic injury after soft coagulation for hemostasis during excision of a peritoneal nodules on the left diaphragmatic surface for examining dissemination. This study was reported in line with the SCARE criteria [8].

## 2 Presentation of case

**Fig. 1:**
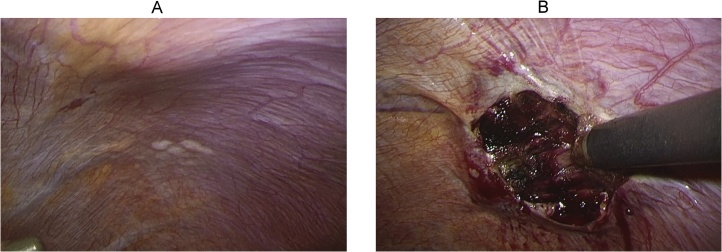
Images of the diaphragmatic peritoneal surface during the first surgery. (A) Three white nodules were found on the diaphragmatic peritoneal surface and were excised for the diagnosis of peritoneal metastasis. (B) Hemostasis after nodule excision.

**Fig. 2:**
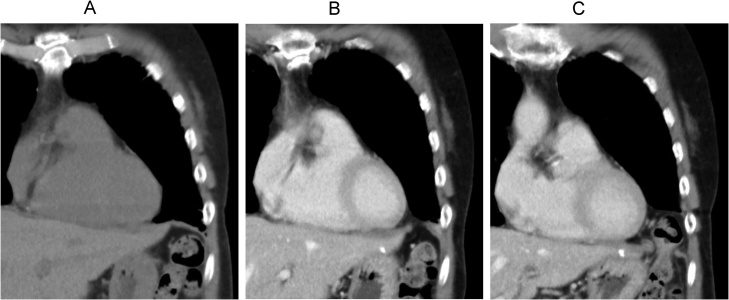
Coronal computed tomography images showing the left diaphragm after the first laparoscopy. (A) At 2 months, the diaphragm seemed intact. (B) At 6 months, a left diaphragmatic hernia was observed. (C) At 9 months, incarcerated splenic flexure of the colon was observed.

**Fig. 3:**
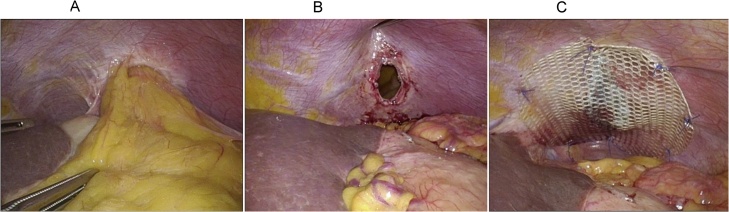
Intraoperative images of the herniation repair during the second surgery. (A) The incarcerated colon. (B) A defect measuring 2 × 2 cm on the left diaphragm. (C) Diaphragmatic hernia repair with a composite mesh.

A 70-year-old woman was diagnosed with gastric gastrointestinal stromal tumor (GIST) and subsequently admitted to the Kyoto University Hospital for laparoscopic partial gastrectomy. Preoperative computed tomography (CT) showed no indication of a diaphragmatic hernia. Intraoperatively, several white nodules were incidentally found on the left diaphragmatic peritoneum; these nodules were excised en bloc using laparoscopic scissors to determine whether they were peritoneal metastatic nodules. Given the presence of hemorrhage at the excision site after excisional biopsy, hemostasis was performed using a soft-coagulation system generated by VIO300D (ERBE Elektromedizin GmbH, Germany), a modern electrosurgical generator (Fig. 1). Pathological examination of the peritoneal nodules suggested that they were not a dissemination of GIST but fibrous tissue. The patient was discharged from our hospital on postoperative day 7 without complications. A follow-up CT scan 2 months after the first laparoscopy revealed a seemingly intact diaphragm (Fig. 2A). However, a follow-up CT scan 6 months after the first laparoscopy revealed a left diaphragmatic hernia (Fig. 2B). No history of any type of abdominal or thoracic trauma was noted during the intervention period. Despite the existence of a diaphragmatic hernia, the patient remained asymptomatic. Thus, she was followed up in an outpatient clinic with careful observation. However, 9 months after the initial surgery, the patient developed postprandial pain in the left upper abdomen. A CT scan revealed exacerbation of the diaphragmatic hernia involving splenic flexure migration into the thoracic cavity (Fig. 2C). The patient subsequently underwent laparoscopic diaphragmatic hernia repair 10 months after the initial diagnostic laparoscopy. Intra-abdominal adhesion was rarely observed, except around the herniated transverse colon through the left-sided diaphragmatic defect. Given that the herniated transverse colon showed no signs of ischemia, it was safely drawn back into the abdominal cavity using laparoscopic graspers. No hernia sac was observed; the diaphragmatic defect was 2 × 2 cm in diameter (Fig. 3A, B). The opening in the diaphragm was closed thoroughly with intracorporeal interrupted suturing using non-absorbable Prolene 3-0 sutures. Thereafter, a composite mesh (Symbotex™, Medtronic, Minneapolis, MN) coated with an antiadhesive material was applied above the suture site and fixed with interrupted suturing using non-absorbable sutures to prevent recurrent hernia (Fig. 3C). The patient recovered well without any complications and was discharged 7 days after the second surgery. Six months after the second surgery, she remained asymptomatic, and a CT scan revealed complete resolution of the diaphragmatic hernia.

## 3 Discussion

Currently, diagnostic laparoscopy for various malignancies, especially advanced gastric cancer, has been widely utilized for the identification of peritoneal disseminated lesions [1,2]. Once suspicious lesions are detected on the peritoneal surface, they are excised for pathological diagnosis. Although excisional biopsy of peritoneal lesions on the diaphragmatic surface has often been performed during diagnostic laparoscopy, diaphragmatic hernias as a late complication have not been reported. This is the first report of an iatrogenic diaphragmatic hernia after diagnostic laparoscopy for peritoneal excision biopsy of the diaphragmatic surface.

Iatrogenic diaphragmatic hernias are a rare complication of abdominal surgery. In the few cases reported previously, iatrogenic diaphragmatic hernias were reported to occur after nephrectomy, gastric banding, total gastrectomy, and adrenalectomy [[4], [5], [6], [7]]; the cause of iatrogenic diaphragmatic hernia is difficult to determine. In the previous reports, the cause of postoperative diaphragmatic hernia has not been elucidated, except in cases where the diaphragm was damaged and needed surgical repair [[4], [5], [6], [7]]. Studies have speculated that the diaphragm could be inadvertently injured intraoperatively through contact with energy devices, such as an electrocautery and ultrasonic coagulation shears, resulting in a delayed diaphragmatic hernia [6,7]. In the present case, the coagulation procedure for hemostasis after peritoneal resection during the first laparoscopic surgery was considered to be the cause of the diaphragmatic hernia. After carefully reviewing the surgery video, heat injury to a part of the diaphragmatic muscle at the time of hemostasis using the soft-coagulation system was retrospectively recognized. The heat generated by soft coagulation might have unexpectedly reached and damaged the deep areas of the diaphragmatic muscle. In addition to the latent heat injury to the diaphragmatic muscle, the pressure gradient between the thoracic and abdominal cavities, which was continuously present within the injured diaphragmatic area, might be a cause of the diaphragmatic hernia.

Soft coagulation was used in the present case considering that it is safe for surrounding tissues because the tissue temperature does not exceed 100 °C unlike other electrocautery coagulation modes [9,10]. We strived to ensure that there was no deep burn injury to the diaphragmatic muscle during hemostasis of the diaphragmatic peritoneum given that the thickness of the diaphragm ranges from 1.65 to 3.70 mm during inspiration and from 1.20 to 2.79 mm during expiration [11]. Nevertheless, despite careful application of the soft-coagulation system, heat may have spread throughout the diaphragmatic muscular layer in the present case. Thus, recognizing that excessive coagulation might lead to unexpected tissue injury, despite soft coagulation, is necessary.

As in the present case, peritoneal excisional biopsy has usually been performed for confirmation of dissemination during laparoscopy and not for therapeutic purposes. Hence, surgeons need to sincerely consider the possibility of delayed severe complications, such as diaphragmatic hernias, following diagnostic laparoscopy. Surgeons should be careful not to perform excessive resection and coagulation. Accordingly, when diaphragmatic muscle damage is suspected during laparoscopic surgery, the injury should be reinforced with sutures.

## 4 Conclusion

We reported a case of delayed-onset iatrogenic diaphragmatic hernia possibly caused by minimal burn injury during hemostasis with soft coagulation after the excision of diaphragmatic surface nodules. Caution should be exercised when applying soft coagulation on the diaphragmatic surface to avoid iatrogenic diaphragmatic hernias. When necessary, diaphragmatic reinforcement should be applied.

## Sources of funding

This research did not receive any specific grant from funding agencies in the public, commercial, or not-for-profit sectors.

## Ethical approval

This study is exempt from ethical approval in our institution.

## Consent

Written informed consent was obtained from the patient for the publication of this report.

## Author contribution

Study concept: Obama K and Hashimoto K.

Data collection: Hashimoto K.

Data analysis and interpretation: Hashimoto K, Obama K, Tsunoda S, Hisamori S and Nishigori T.

Writing the paper: Hashimoto K and Obama K.

Reviewing the paper: Tsunoda S, Hisamori S, Nishigori T, Sakaguchi M, Ueda Y, Nakanishi N and Sakai Y.

All authors have read and approved the final manuscript.

## Registration of research studies

Because this article is a case report, it is not applicable to be registered in a publicly accessible database.

## Guarantor

Kazutaka Obama.

## Provenance and peer review

Not commissioned, externally peer-reviewed.

## Declaration of Competing Interest

No potential conflicts of interest exist.
